# Früherkennung primärer Sprachentwicklungsstörungen – zunehmende Relevanz durch Änderung der Diagnosekriterien?

**DOI:** 10.1007/s00103-022-03571-6

**Published:** 2022-07-21

**Authors:** Christiane Kiese-Himmel

**Affiliations:** Phoniatrisch/Pädaudiologische Psychologie, Institut für Medizinische Psychologie und Medizinische Soziologie, Universitätsmedizin Göttingen, Georg-August-Universität Göttingen, Waldweg 35, 37075 Göttingen, Deutschland

**Keywords:** Sprach- und Sprechentwicklungsstörung, Umschriebene Entwicklungsstörung des Sprechens und der Sprache, Sprachentwicklungsstörung, Früherkennung, Public Health, Language and speech development disorder, Specific language impairment, Developmental language disorder, Early detection, Public health

## Abstract

Sprachentwicklungsstörungen (SES) sind die häufigsten Entwicklungsstörungen im Kindesalter. Die „primären SES“ (Prävalenz etwa 7 %) werden im Unterschied zu „sekundären SES“ nicht durch andere Entwicklungsstörungen bzw. Erkrankungen (mit-)verursacht. In der deutschen Modifikation der Internationalen Statistischen Klassifikation der Krankheiten und verwandter Gesundheitsprobleme (ICD-10-GM-22) werden die primären SES als „Umschriebene Entwicklungsstörungen des Sprechens und der Sprache“ (USES) bezeichnet, wobei u. a. ein Intelligenzquotient (IQ) < 85 als Ausschlusskriterium gilt. In der ICD-11 werden primäre SES als „Developmental Language Disorder“ (DLD) angegeben.

Deutschsprachige Sprachtherapeut*innen möchten nun den Terminus „USES“ durch „DLD“ ersetzen und dabei die Diagnosekriterien verwenden, die vom internationalen CATALISE-Consortium (Criteria and Terminology Applied to Language Impairments Synthesizing the Evidence) vorgeschlagen werden, in deren Bestreben, eine Neubestimmung des Störungsbildes vorzunehmen. Nach dieser Konzeption werden jedoch nur Kinder mit einer geistigen Behinderung (IQ < 70) von der Diagnose ausgeschlossen. Dieser Wechsel in den diagnostischen Kriterien hätte höchstwahrscheinlich einen Anstieg der Prävalenz zur Folge. Damit wird die Frage der Früherkennung wichtiger denn je. In diesem Diskussionsbeitrag wird erklärt, dass die Public-Health-Relevanz der primären SES wächst und systematischen Früherkennungsuntersuchungen eine noch wichtigere Rolle zukommen wird. Mit einer frühzeitigen Diagnose und Therapie können Risiken in den Bereichen psychische Gesundheit, Verhalten und Entwicklung von Fertigkeiten gemindert werden.

Derzeit erfolgen Diagnostik (und Therapie) meist relativ spät. Der Ausweg könnte in der Anwendung neurobiologischer Parameter liegen. Dazu werden jedoch weitere Studien benötigt, die Kinderkohorten in einem prospektiven longitudinalen Design auf Frühindikatoren untersuchen. Auch die Bildung eines Früherkennungsindex aus mehreren Indikatoren ist zu erwägen.

## Abstract (en)

Language development disorders (in German: Sprachentwicklungsstörungen, SES) are the most common developmental disorders in childhood. In contrast to “secondary SES,” “primary SES” (prevalence about 7%) are not (co-)caused by other developmental disorders or diseases. In the German modification of the International Statistical Classification of Diseases and Related Health Problems (ICD-10-GM-22), primary SES are referred to as “circumscribed developmental disorders of speech and language” (in German: USES; international previously known as Specific Language Impairment SLI), with an intelligence quotient (IQ) < 85 as an exclusion criterion, among other criteria. In ICD-11, primary SES are listed as “developmental language disorders” (DLD).

German-speaking speech and language therapists would now like to replace the term “USES” with “DLD” using the diagnostic criteria proposed by the international CATALISE consortium (Criteria and Terminology Applied to Language Impairments Synthesizing the Evidence), in an effort to redefine the disorder. However, according to this conceptualization, only children with an intellectual disability (IQ < 70) would be excluded from the diagnosis. This change in the diagnostic criteria would most likely result in an increase in prevalence of DLDs. This makes the issue of early detection more important than ever. This discussion paper explains that the public health relevance of primary SES is growing and that systematic early detection examinations will play an even more important role. With early diagnosis and treatment, risks in the areas of mental health, behaviour and skill development can be mitigated.

Currently, diagnosis (and therapy) are usually carried out relatively late. The way out could lie in the application of neurobiological parameters. However, this requires further studies that examine child cohorts for early indicators in a prospective longitudinal design. The formation of an early detection index from several indicators should also be considered.

## Einleitung

Vor gut 10 Jahren starteten Wissenschaftler*innen aus Australien und dem Vereinigten Königreich eine Initiative, um Sprachentwicklung und ihre Störungen zu einem Thema von Public Health zu machen [1–3]. Auch in Deutschland sind Gesundheit und Entwicklung von Kindern und Jugendlichen seit vielen Jahren ein wichtiges Thema. Dennoch werden die Sprachentwicklung und ihre Störungen weiterhin fast ausschließlich als ein individuelles Problem betrachtet, das entsprechend auch individuell zu behandeln ist. Das medizinische Fachgebiet zur Diagnostik, Behandlung, Vorbeugung und Rehabilitation von Störungen der menschlichen Kommunikation, die Phoniatrie und Pädaudiologie, sind wie die Logopädie/Sprachtherapie als therapeutische Dienstleister auf den Einzelfall („One-to-one-Service“) ausgerichtet. Auf gesellschaftlicher Ebene erweist es sich aber als zunehmend wichtig, Änderungen auf breiter Ebene anzustoßen, das meint, etwaige Probleme in der Sprachentwicklung bereits im Vorfeld zu erkennen.

Im Zuge einer Neuausrichtung von Public Health haben sich – in Abgrenzung zur kurativen Medizin – Krankheitsprävention und Gesundheitsförderung zu Säulen gesundheitspolitischer Maßnahmen entwickelt. Das rechtzeitige Erkennen von Sprachentwicklungsstörungen (SES) und das Einleiten geeigneter Interventionsmaßnahmen im Zuge einer umfassenden Betrachtung der Gesundheit betroffener Kinder entspricht diesem Verständnis. Die Genese von SES, ihre gelungene oder misslungene Bewältigung, strukturelle Merkmale therapeutischer Hilfsangebote und nicht zuletzt die Aufmerksamkeit der familiären und sozialen Umgebung prädestinieren Sprachentwicklung zu einem Public-Health-Thema.

Im vorliegenden Beitrag wird auf „primäre SES“ fokussiert, also auf solche Sprachentwicklungsstörungen, die nicht durch andere Entwicklungsstörungen bzw. Erkrankungen (mit-)verursacht sind (im Gegensatz zu „sekundären SES“). Primäre SES sind durch anhaltende Defizite beim Erwerb, Verstehen, Produzieren bzw. Gebrauch von Sprache gekennzeichnet, die Sprachleistung eines Kindes liegt dabei deutlich unter dem, was angesichts dessen Lebensalters zu erwarten wäre. Primäre SES sind genetisch bedingt bei individuell unauffälligen somatischen und Umweltgegebenheiten. Dabei ist die Sprachentwicklung eines Kindes von Geburt an beeinträchtigt, also schon in der präverbalen Entwicklungsphase. Liegt eine primäre SES bei mehrsprachig aufwachsenden Kindern vor, so betrifft sie jede Sprache, mit der ein Kind aufwächst. Primäre SES treten mit einer Prävalenz von etwa 7 % [2, 4, 5] relativ häufig auf. Jungen sind vermehrt betroffen [4]. Prävalenzschätzungen in populationsbasierten Studien fallen geringfügig höher aus, z. B. 7,58 % [6].

Über die präzise Definition und die Terminologie von primären SES ist in den letzten Jahren ein wissenschaftlicher und klinischer Disput entstanden. Dabei geht es um Merkmale und Kriterien der Definition, aber ebenso um diagnostische und therapeutische Konsequenzen. Der vorliegende Beitrag möchte darauf aufmerksam machen, dass die Diagnose einer primären SES aufgrund einer geplanten definitorischen Erweiterung zukünftig häufiger gestellt werden könnte, wodurch auch die Public-Health-Relevanz der Erkrankung weiter ansteigt.

In diesem Diskussionsbeitrag werden zunächst die gültigen Definitionen von primären SES gemäß der Internationalen Statistischen Klassifikation der Krankheiten und verwandter Gesundheitsprobleme (ICD-10-GM-22 [7], ICD-11 [8]) und gemäß englischsprachigem CATALISE-Consortium (**C**riteria **A**nd **T**erminology **A**pplied to **L**anguage **I**mpairments **S**ynthesizing the **E**vidence) vorgestellt. Darauffolgend werden die derzeitigen Möglichkeiten der Früherkennung einer primären SES in der Praxis sowie ihre Public-Health-Relevanz aufgezeigt. Schließlich wird unter Verweis auf neuroanatomische Forschungsergebnisse die Notwendigkeit innovativer Forschung zur Früherkennung herausgestellt.

### Termini und Definitionen

Eine primäre SES wird in der aktuell verwendeten deutschen Modifikation der ICD-10 als „Umschriebene Entwicklungsstörung der Sprache“ (USES; ICD-10-GM-22: Code F80) bezeichnet (F80.1: expressiv; F80.2: rezeptiv). Im englischsprachigen Raum wird die USES seit den 1980ern auch „Specific Language Impairment“ (SLI) genannt, eine Bezeichnung, die mit dem Namen „Spezifische Sprachentwicklungsstörung“ teilweise in den deutschsprachigen Raum übernommen wurde.

Eine USES zeigt sich in der frühen Entwicklung durch wesentliche zeitliche und inhaltliche Abweichungen vom normalen Muster des Sprach- und Sprecherwerbs – *bei altersentsprechender Allgemeinentwicklung.* Das bedeutet unter anderem, dass neurologische Erkrankungen, Hörstörungen und eine Intelligenzminderung mit einem Intelligenzquotienten (IQ) < 85 als Ausschlusskriterien gelten. Das Risiko für eine Beeinträchtigung der psychischen Gesundheit, für Verhaltensprobleme sowie für die Ausbildung umschriebener Entwicklungsstörungen schulischer Fertigkeiten ist hoch, weswegen zwingend eine frühe Diagnostik indiziert ist, basierend auf Ausschluss‑, Entwicklungs- und Diskrepanzkriterien. Das bedeutet: (1) Erkrankungen, Entwicklungsstörungen, Umgebungsbedingungen für die Sprachprobleme auszuschließen; (2) eine individuelle Sprachleistung von mindestens 1,5 Standardabweichungen unter der Altersnorm in altersadäquaten standardisierten und normierten Sprachtests nachzuweisen; (3) eine bedeutende Differenz (von mindestens 1 Standardabweichung) zwischen den derart erhobenen Sprachleistungen und der nonverbalen Intelligenzhöhe als Indikator für das allgemeine Intelligenzniveau zu belegen. Durch den letztgenannten Nachweis soll sichergestellt sein, dass der Sprachentwicklungsstand eines Kindes bedeutsam unterhalb seines allgemeinen Intelligenzniveaus liegt.

Viele Kinder mit USES haben Probleme, Sprache in sozialen Situationen richtig einzusetzen. Es fällt ihnen möglicherweise schwer, z. B. mit Klassenkamerad*innen, Erzieher*innen, Lehrer*innen, in Kontakt zu treten, über ihre Gefühle zu sprechen [6], sich in einem Gespräch miteinander abzuwechseln, beim Thema zu bleiben oder lange Sätze zu verstehen. Auch kann es ihnen schwerfallen, Informationen verbal weiterzugeben und Geschichten zu erzählen [8]. Die Schwierigkeit, anderen ein Problem verbal verständlich machen zu müssen, führt mitunter dazu, dass sich die Kinder frustriert oder wütend fühlen und auf eine unangemessene Art und Weise handeln. Die Störung ist therapiebedürftig [9, 10]. Der Verlauf einer USES ist stetig, ohne Spontanremission. Trotz Therapie können Sprachdefizite bis ins Erwachsenenalter persistieren.

Die ICD-11 [8], die bereits am 01.01.2022 in Kraft getreten ist, jedoch noch einige Jahre bis zu ihrer Etablierung benötigen wird, bis der kontinuierliche Qualitätssicherungsprozess abgeschlossen ist, enthält nun die Kategorie „Developmental Speech or Language Disorder“ (DLD; Oberkategorie 6A01). In diese fallen Sprach- und Kommunikationsprobleme in der frühen Kindheit, die über die Grenzen der normalen Abweichung im Vergleich zu Alter und Intelligenzniveau eines Kindes hinausgehen; sie sind ursächlich nicht mit sozialen oder kulturellen Faktoren (z. B. regionaler Dialekt) assoziiert und nicht durch eine Sinnesbehinderung, neurologische Entwicklungsstörung, Auswirkungen einer Hirnverletzung oder zerebralen Infektion zu erklären. Unterschieden werden: DLD mit Beeinträchtigung von rezeptiver und expressiver Sprache (6A01.20); DLD mit Beeinträchtigung von hauptsächlich expressiver Sprache (6A01.21); DLD mit Beeinträchtigung von vorwiegend pragmatischer Sprache (Kommunikation; 6A01.22) und DLD mit einer anderen spezifischen Sprachentwicklungsbeeinträchtigung (6A01.23).

Eine Gruppe von deutschsprachigen Sprachtherapeut*innen (z. B. Logopäd*innen; akademische Sprachtherapeut*innen; klinische Linguist*innen; Sprachheilpädagog*innen) möchte nun, dass der Terminus „USES“ durch den Terminus „DLD“ ersetzt wird [11]. Dabei sollen Diagnosekriterien verwendet werden, die vom CATALISE-Consortium 2016 und 2017 vorgeschlagen wurden [12, 13]. Bei dieser Gruppe handelt es sich wiederum um einen Zusammenschluss von 59 internationalen englischsprachigen Expert*innen aus Wissenschaft, mehrheitlich sprachtherapeutischer, aber auch ärztlicher, psychologischer und pädagogischer Praxis aus dem Vereinigten Königreich, Hongkong, USA, Kanada, Australien, Neuseeland, Irland. Ihr DLD-Konzeption schließt auch Kinder mit ein, die neben Sprachproblemen diskrete neuronale Irregularitäten ohne klare biomedizinische Ätiologie haben, etwa eine Hörminderung durch wiederkehrende Otitiden und die somit eine Minderung ihrer Jahreshörbilanz aufweisen oder koexistente neurologische Funktionsschwächen wie eine auffällige Lateralisierung, Auffälligkeiten in den Exekutivfunktionen, im phonologischen Arbeitsgedächtnis, in der Wahrnehmung oder Motorik.

Allein die Tatsache, dass solche Auffälligkeiten häufig bei Kindern mit primären SES zu beobachten sind, deutet nach Meinung von CATALISE darauf hin, dass für diese Klientel ein breiterer Phänotyp zutreffender wie auch das enge Verständnis einer USES (bzw. SLI) artifiziell ist. So könnten Kinder mit einer unterdurchschnittlichen nonverbalen Intelligenz durchaus miteingeschlossen werden. Insbesondere soll nicht am Exklusionskriterium IQ < 85 festgehalten werden, sondern es sind nur Kinder auszuschließen, die das Kriterium einer geistigen Behinderung erfüllen (IQ < 70). Gemäß CATALISE wäre dies eine „differenzierende Bedingung“ („differentiating condition“). Für den Fall, dass die Sprachentwicklungsauffälligkeiten Bestandteil einer komplexen Störung sind, würde die Diagnose demnach erweitert werden zu: „SES, assoziiert mit [verursachender Faktor]“, also z. B. „SES, assoziiert mit Down-Syndrom (Trisomie 21)“.

Zudem sollen „Begleiterscheinungen“ („co-occuring conditions“) ohne kausalen Bezug (z. B. Aufmerksamkeitsdefizit-Hyperaktivitätsstörung (ADHS)) und Risikofaktoren („risk factors“) unterschieden werden. Letztere sind solche, für die eine positive statistische Korrelation mit Sprachstörungen (ebenfalls ohne Kausalität) belegt ist. Das Diskrepanzkriterium zum nonverbalen IQ gemäß ICD-10 wird abgelehnt. Obwohl die CATALISE-Festlegungen die Verwendung von standardisierten Tests zur Identifizierung von DLD nicht infrage stellen, legen sie mehr Wert auf die Auswirkungen der Störung hinsichtlich sozialer und Schulleistungen als auf niedrige Sprachgrenzwerte. Statt Orientierung an statistisch definierten Grenzwerten in standardisierten und deutschsprachig normierten Tests sei vor allem der Schweregrad funktioneller Auswirkungen hervorzuheben.

An der Terminologie nach der CATALISE-Konzeption und den hiermit verbundenen Implikationen haben psychologische und medizinische Berufsgruppen, die in ihrem Arbeitsalltag mit sprachentwicklungsgestörten Kindern zu tun haben, deutliche Kritik formuliert [14–17]. Der Austausch des Ansatzes „Komorbidität“ durch „verursachende Faktoren“, „Risikofaktoren“ und „Begleiterscheinungen“ könnte eine Unterlassung von Differenzialdiagnostik bedeuten. Unter anderem bestünde die Gefahr, ätiologisch bedeutsame Klassifikationen und differenzialdiagnostische Grenzen zu verwischen und auf ärztliches und psychologisches Fachwissen in Diagnostik und Therapie von SES im Kindesalter zu verzichten [16].

Die geplante Neuausrichtung von Terminologie und Definition löste eine interdisziplinäre Online-Delphi-Befragung^1^ von Expert*innen zu „Definition und Nomenklatur von Störungen der Sprache im Kindesalter im typischen Zeitraum der Sprachentwicklung“ aus, organisiert und moderiert von der Gesellschaft für Interdisziplinäre Spracherwerbsforschung und Kindliche Sprachstörungen im Deutschsprachigen Raum (GISKID). Die erste Expert*innenbefragung unter registrierten Teilnehmer*innen aus verschiedenen sachrelevanten Berufsgruppen im deutschsprachigen Raum ist abgeschlossen; an ihr nahmen 212 Sprachtherapeut*innen/Logopäd*innen, 142 Ärzt*innen, 40 (Sonder‑)Pädagog*innen, 31 Linguist*innen/Sprechwissenschaftler*innen, 21 Psycholog*innen und 3 Neurowissenschaftler*innen aus deutschsprachigen Ländern teil. Es bestand Einigkeit darüber, dass eine einheitliche Terminologie in allen deutschsprachigen Ländern und Professionen wünschenswert ist. Auf Basis der Ergebnisse der ersten sowie einer weiteren standardisierten Onlinebefragung (an der weniger Expert*innen teilnahmen) fand eine dritte elektronische Befragung statt. In den nächsten Monaten sollen alle Delphi-Runden ausgewertet, evaluiert und publiziert werden. Die Teilnahmerate nahm über die 3 Delphi-Runden ab (Drop-outs), war aber jedes Mal quantitativ von Sprachtherapeut*innen/Logopäd*innen dominiert, obgleich auch unter ihnen Drop-outs waren. Trotz einer elektronischen und damit eher einfach zu handhabenden ökonomischen Befragung scheint die Bereitschaft zur Teilnahme, zumindest für niedergelassene ärztliche und psychologische Expert*innen, tendenziell niedriger gewesen zu sein; die Befragten waren vor allen Sprachtherapeut*innen/Logopäd*innen, was möglicherweise bedeuten kann, dass sich deren Einschätzungen letztendlich leichter durchsetzen.

Da internationale Wissenschaftler*innen in Publikationen den Terminus „DLD“ verwenden (z. B. [18–22]), sind ggf. negative Konsequenzen zu erwarten, wenn dieser in deutschsprachigen Ländern sensu CATALISE eingeführt werden sollte, da die ICD-11 zur Morbiditätskodierung in Deutschland noch mehrere Jahre in Anspruch nehmen wird (sie liegt erst in einer Entwurfsfassung vor). Zuerst einmal dürfte es zu einem Anstieg der „administrativen“ Prävalenz von primären SES (und infolgedessen auch zu einem Anstieg von Sprachtherapieindikationen) kommen, ohne dass diese immer gerechtfertigt wären. Weil man um die Bedeutung einer frühen Erkennung dieser häufigen Entwicklungsstörung weiß, die im Einzelfall durchaus eine lebenslängliche Belastung darstellen kann, dürften fast reflexhaft auch die Präventionsbemühungen zunehmen, die dann aber bei den falsch klassifizierten SES ins Leere greifen und unnötige Kosten verursachen.

### Früherkennung von primären SES

Derzeit wird in der Frühdiagnostik als prädiktiver Indikator der „expressive Wortschatz“ eingesetzt, der sprachentwicklungsverzögerte Kinder (Late Talkers) detektiert, doch sprachentwicklungsverzögerte Kinder sind nicht zwingend „sprachentwicklungsgestört“. Der expressive Wortschatz wird mit standardisierten, psychometrisch abgesicherten Untersuchungsinstrumenten erhoben (ab dem 18. Lebensmonat bis zum 26. bzw. 30. Monat) – also eher relativ spät aus der Perspektive von Neuroplastizität. Die Mehrzahl der deutschsprachigen sprachdiagnostischen Testverfahren ist für das Kindergarten- und Vorschulalter konstruiert und scheidet daher als frühes Früherkennungsmaß aus; zudem verfügen diese Tests meistens nicht über Maßzahlen zu Sensitivität und Spezifität, die die Beurteilung ihrer Güte ermöglichen. Deswegen fehlt auch ein positiver Likelihood-Ratio (Quotient von Sensitivität geteilt durch (1-Spezifität)); ein solcher beschreibt die Wahrscheinlichkeit für ein positives Ergebnis eines betroffenen Kindes (richtig-positiv) in Relation zur Wahrscheinlichkeit eines positiven Ergebnisses bei einem gesunden Kind (falsch-positiv).

Die Sprachentwicklung ist ein komplexes Zusammenspiel von biologischen, kognitiven und Umgebungsfaktoren. In Anbetracht der hohen Variabilität von Sprech- und Sprachentwicklung bzgl. der einzelnen linguistischen Parameter [23] lässt sich eine primäre SES bislang nicht vor dem 3. Geburtstag eines Kindes reliabel diagnostizieren [9, 10]. Aus der Fachliteratur sind zwar singuläre Risikofaktoren bekannt (wie familiäre Disposition; männliches Geschlecht; Frühgeburt; Wortverständnisdefizit im 1. Lebensjahr; die Bildung weniger Konsonanten; Entwicklungsverzögerung in der Gestenproduktion; fehlende Spontanimitation; verspäteter Sprechbeginn; expressiver Wortschatzumfang < 50 Wörter mit 24 Monaten; fehlende 2‑Wort-Kombination bis zum 30. Lebensmonat), doch, wie oben ausgeführt, ist selbst der verspätete Sprechbeginn (Late Talking) bei jedem 5. Kind kein eindeutiger Risikofaktor, da viele Late Talkers ihren Rückstand bis zum 3. Geburtstag aufgeholt und ihre Altersgefährten sprachlich eingeholt haben (Late Bloomers). Ein Konsensus zur Rangreihe der prädikativen Kraft früher SES-Indikatoren fehlt; auch ist nicht auszuschließen, dass erst mehrere Indikatoren zusammengenommen eine primäre SES bedingen.

Wenn also eine primäre SES, die immer genetisch angelegt ist, nicht früh erkannt und von einer effektiven therapeutischen Intervention begleitet wird, bleiben individuelle Entwicklungspotenziale bzw. Entwicklungsressourcen eines derart gestörten Kindes ungenutzt, da die neuronale Plastizität, insbesondere die synaptische Plastizität als Basis für Lern- und Gedächtnisprozesse, im Kleinkindalter besonders hoch ist (z. B. [24, 25]). Somit scheint es nur schlüssig und dringend geboten – und zwar nicht nur für den Fall einer Änderung von Definitions- und Diagnosekriterien – das Konzept „Früherkennung“ für Kinder mit primärer SES stärker in den Fokus wissenschaftlicher Bemühungen zu stellen.

Im Folgenden werden Möglichkeiten der Früherkennung genannt. Hiernach werden exemplarisch einige entwicklungsneurologische Studien genannt, die nach Indikatoren für das Risiko einer primären SES an definierten Kinderklientelen suchten und denen zukünftig Bedeutung in der Früherkennung zukommen könnte.

Kiese-Himmel [30] schlug 1999 3 Verhaltensmarker zur Früherkennung vor, die laut entwicklungspsychologischer Fachliteratur als valide Frühindikatoren einer USES empirisch bestätigt worden waren: (1) Qualität der frühen Mutter-Kind-Interaktion; (2) haptische Exploration von Objektqualitäten, inkl. der hierfür verwendeten Explorationsprozeduren; (3) expressiver Wortschatz in der frühen lexikalischen Entwicklung. In der klinischen Praxis hat sich aus zeitökonomischen Gründen die Erhebung des expressiven Wortschatzes durchgesetzt. Sie erfolgt mittels Befragung von Eltern/unmittelbaren Bezugspersonen anhand standardisierter Wortschatzchecklisten im Alter von 12 Monaten [31] oder 18 Monaten (z. B. [32, 33]). Ein solches Vorgehen, wenngleich ökonomisch, ist aber nicht fehlerfrei, da ein Risiko der Verfälschung durch die subjektive (Laien‑)Beurteilung besteht, sodass sich z. B. die soziale Erwünschtheit auf die Antworten auswirkt. Dies kann die Reliabilität der Früherkennung beeinträchtigen. Zudem erscheint es wichtig, Indikatoren zu finden, die noch früher in der Kindesentwicklung ansetzen.

#### Auf der Suche nach Frühindikatoren

##### Protophone.

Erstrebenswert ist ein Frühindikator im präverbalen Entwicklungsabschnitt. Protophone (frühe Vokalisationen) gelten als Vorläufer von Sprache, weil sie die Entwicklung der akustischen Merkmale von Sprachlauten aufzeigen, ihre Produktion ist weitgehend endogen bedingt. Schon im ersten Lebensmonat kommen sie häufiger vor als Schreien, selbst bei Frühgeborenen [34]. Die Anzahl der Protophone, die ein Säugling produziert, ist erstaunlich groß; sie wurde durch Codierung zufällig ausgewählter Stichproben aus ganztägigen Aufnahmen auf etwa 3500 am Tag geschätzt, eine Zahl, die im Laufe des ersten Lebensjahres kaum schwankt. In der 2. Hälfte des 1. Lebensjahres tritt in der präverbalen Sprachentwicklung kanonisches Lallen auf: Konsonant plus Vokal werden zu Silben verbunden (wie „ba“; „da“; „ma“), zu duplizierten Silben (wie „baba“; „dada“; „mama“ bzw. Lallwörtern wie „bababa“, die lediglich einem Wort ähneln, aber nicht intentional produziert werden). Häufigkeit und Variabilität kanonischer Vokalisation sind mit der Produktion erster Wörter assoziiert. Frühe Vokalisationen vermögen jedoch nicht die rezeptive Sprachentwicklung vorherzusagen [35].

In einem narrativen Review über retro- und prospektive Studien bewerteten Lang et al. [36] das Potenzial des kanonischen Lallens, also die Fähigkeit zur Bildung wohlgeformter Silben mit temporalen und spektralen Eigenschaften der Erwachsenensprache als (prädiagnostischen) Indikator zur Früherkennung von „spät erkannten Entwicklungsstörungen“ wie Autismusspektrumstörung (ASS), Rett- und fragiles X‑Syndrom, die mit Sprachentwicklungsbeeinträchtigungen einhergehen. Ihr Fazit: Kanonisches Lallen eignet sich nicht als Frühindikator. Lang et al. [36] empfehlen zur Früherkennung von präverbalen Entwicklungsabweichungen eine detaillierte linguistisch-phonetische Analyse der frühen Vokalisationen (inkl. akustischer Signalanalyse) im Längsschnitt. Übertragen auf die Früherkennung primärer SES im präverbalen Entwicklungsabschnitt ist zu vermuten, dass eine phonetische Analyse ontogenetisch früher Vokalisationen aussagekräftiger ist als die reine Feststellung des Vorhandenseins von Protophonen.

##### Hirnstrukturen.

Vassar et al. [26] untersuchten mittels Magnetresonanztomographie (MRT) die Hirnstruktur im Hinblick auf die frühe Sprachentwicklung bei 92 sehr früh geborenen Kindern (Geburtsgewicht ≤ 1500 g; Gestationsalter ≤ 32 Wochen) im korrigierten Alter von 18–22 Monaten. Die Mikrostruktur der weißen Substanz wurde mit der Diffusionstensorbildgebung (DTI = diffusionsgewichtetes MRT) beurteilt. 31 von 92 Kindern erreichten im Gesamtsprachentwicklungswert in den Bayley Scales of Infant-Toddler Development-III [27] einen Standardwert < 85, darunter 15 von 92 einen Wert < 70, was auf eine mittlere bis schwere Entwicklungsverzögerung hindeutet. Kinder mit einer Kleinhirnasymmetrie hatten signifikant niedrigere rezeptive Sprachwerte (*p* = 0,016). Multivariate Modelle identifizierten 3 Hirnregionen, die die Sprachleistungen am besten prognostizieren. Eindeutige logistische Regressionsmodelle sagten Hochrisikokinder voraus, definiert durch Sprachwerte, die mehr als eine Standardabweichung unter dem Durchschnitt lagen. Mit hoher Genauigkeit wurden der expressive, der rezeptive (100 % Sensitivität; 90 % Spezifität) und der zusammengesetzte Sprachentwicklungswert (89 %; 86 %) vorhergesagt.

Ähnliche Ergebnisse bei sehr früh Geborenen erzielten Bugada et al. [28] in einer Pilotstudie an einem kleinen Kollektiv. Die Bahnen der weißen Substanz wurden mithilfe der DTI-ermittelten Parameter „mittlere Diffusivität“ und „fraktionelle Anisotropie“^2^ bewertet. Die fraktionelle Anisotropie des inferioren longitudinalen Fasciculus (dieses Faserbündel verbindet Temporal- und Okzipitallappen) war in univariaten Analysen mit den Bayley-III-Werten [27] assoziiert und erwies sich in multivariaten Analysen als ein unabhängiger Prädiktor für die kognitive und sprachliche Entwicklung in Bayley-III. Die Einbeziehung neuer Biomarker wie der fraktionellen Anisotropie des inferioren longitudinalen Fasciculus in strukturelle MRT-Befunde könnte die Genauigkeit der Modelle zur Vorhersage der neuronalen Entwicklung verbessern.

Ähnliche methodische Studien, z. B. an 89 Frühgeborenen [29], fanden, dass die Kombination von perinatalen klinischen Informationen und neonatalen DTI-Messungen der Mikrostruktur der weißen Substanz zu einer genauen Vorhersage der Sprachentwicklung nach 2 Jahren (bei korrigiertem Gestationsalter) führt. Das heißt, es werden solche Kinder erkannt, die wahrscheinlich Sprachdefizite entwickeln werden.

### Public-Health-Relevanz der Früherkennung von primären Sprachentwicklungsstörungen

Der Gebrauch der Terminologie von primären SES ist offensichtlich divergent und die aktuelle Terminologie- und Definitionsdebatte zeigt, dass es leider keine professionsübergreifende, von allen Expert*innen anerkannte Klassifikation ihrer Symptome und Erkrankungen gibt. Unbestritten ist eine primäre SES eine Multi-Deficit Disorder. Während im Konzept der USES (ICD-10-GM-22, Code F80.-) tendenziell eher latente kognitive Schwächen eines Kindes toleriert werden, schließt die CATALISE-Konzeption „DLD“ auch gravierende multiple Defizite ein, da eine unterdurchschnittliche Intelligenz nun nicht mehr ein Ausschlusskriterium für die Diagnose ist. Allerdings ist davon auszugehen, dass die bevorzugte Terminologie in Abhängigkeit von der Art der fachlichen Ausbildung eines Experten/einer Expertin variiert [37]. Diagnostische Labels sind ein wichtiges Instrument, um das Verständnis für Sprachentwicklungsprobleme bei Kindern zu fördern sowie angemessene Therapie und ggf. weiteren Support bereitzustellen. Dies kann aber nur geschehen, wenn die beteiligten Professionen einen Konsens in Definition und Label haben, also „dieselbe Sprache sprechen“.

Bislang klammerte die Terminologie- und Definitionsdebatte die bei Einführung des Terminus DLD möglichen negativen Konsequenzen weitgehend aus: z. B. die mutmaßliche Erhöhung der Prävalenz und infolgedessen eine Zunahme an Verordnungen für sprachtherapeutische Interventionen. Mit einer Prävalenzerhöhung aufgrund erweiterter Definitions- und diagnostischer Kriterien einer DLD wird aber die Notwendigkeit der standardisierten Früherkennung anhand valider und reliabler Indikatoren aus klinischer Sicht wichtiger denn je. Doch praktikable valide Frühindikatoren auf Verhaltensebene in Ergänzung zu einer somatischen Frühdiagnostik fehlen bislang. Eventuell könnte sich hierfür die Analyse der Produktion von Protophonen anbieten, welche eine hohe Bedeutung für den Erwerb von Lautsprache haben, doch Protophone wurden nicht in populationsbasierten Studien im Längsschnitt auf ihre diagnostische Bedeutung hin untersucht.

Die Sachlage zur Früherkennung von Kindern mit primären SES, aufgehängt an der nicht auszuschließenden Übernahme der CATALISE-Konzeption „DLD“ im deutschsprachigen Raum und der hieraus vermutlich resultierenden Prävalenzerhöhung, bedeutet eine Herausforderung für den Bereich Public Health. Public-Health-Themen zielen auf *vorrangige* Gesundheitsprobleme [38], die beispielsweise dadurch gekennzeichnet sind, dass sie (a) häufig auftreten (Störungen der primären Sprachentwicklung gehören zu den häufigsten Gesundheitsproblemen von Kindern [39]), (b) schwerwiegende Folgen haben können (Sprache und Kommunikation sind essenzielle Voraussetzungen für eine erfolgreiche Schul- und Lernkarriere und Einstieg in das Berufsleben (z. B. [2])) und/oder (c) hohe Kosten verursachen (geringe Sprachkenntnisse und Sprachstörungen verstellen Bildungschancen und bürden der Gesellschaft und Wirtschaft direkte und indirekte Kosten auf [1]).

Die Relevanz der Früherkennung von primären SES ist in den letzten Jahren u. a. an der hohen Zahl von Sprachtherapien abzulesen, die meist nach dem 3. Geburtstag verordnet werden. USES (ICD-10-GM-22, Code F80) waren laut der Heilmittelberichte des Wissenschaftlichen Instituts der AOK in den letzten 3 Jahren die häufigste Diagnose bei sprachtherapeutischen Patienten*innen im Kindesalter. So dominierten USES-Diagnosen unter AOK-versicherten sprachtherapeutischen Patient*innen im Jahr 2018 mit 55,2 % [40], 2019 [41] waren es 56,1 % und 2020 [42] bereits 57,0 %. Daher erscheint eine Verlagerung des Schwerpunkts von einer allein klinischen Versorgung des Kindes zu einer Versorgung nach Public-Health-Grundsätzen sinnvoll.

Public Health bietet einen methodischen Ansatz, um Gesundheitsprobleme zu erforschen, und einen Werkzeugkasten, um diese evidenzbasiert zu verändern. Wenn es also vernünftig erscheint, Probleme der Sprachentwicklung im Rahmen eines Public-Health-Ansatzes zu erforschen und zu verstehen, dann müssen wir, die wir professionell in diesem Gebiet arbeiten, damit beginnen. Exemplarisch und stellvertretend sei nur eine Voraussetzung für einen fruchtbaren Public-Health-Ansatz zum Thema Sprachentwicklung und ihre Störungen genannt: epidemiologisch verlässliche Daten. Nicht selten berichten Kliniken oder Praxisverbünde über ihre Klientel und versuchen daraus Prävalenzen für bestimmte Störungsbilder abzuleiten. Zumeist fehlen dabei die 3 wichtigsten Voraussetzungen: die Kenntnis der Basispopulation, eine einheitliche wissenschaftlich verbindliche Definition der Störung und eine repräsentative Erhebung. Auch hieran ist abzulesen, dass eine verbindliche Störungsdefinition das Fachgebiet „Public Health“ tangiert.

In Zukunft werden Studien benötigt, die z. B. mit nichtinvasiver diffusionsgewichteter MR-Bildgebung und „klassischen“ MRT-Methoden in sehr jungem Kindesalter durchgeführt werden, um valide und reliable Indikatoren zur standardisierten Früherkennung zu finden, denn unser Wissen über die mit primärer SES verbundenen Hirnanomalien ist begrenzt. Hiernach sind Kinderkohorten im prospektiven longitudinalen Design auf Frühindikatoren, die sich in solchen experimentellen Studien als geeignet erwiesen, zu untersuchen, etwa auf frühe hirnstrukturelle Korrelate, die für Probleme bei der Verarbeitung und Speicherung von gehörter Sprache verantwortlich sind. Im nächsten Schritt sind zur Früherkennung geeignete Maße an größeren Kohorten zu validieren. Auch die Bildung eines Früherkennungsindex aus mehreren Indikatoren zu einer neuen Variablen ist zu erwägen, sofern alle relevanten Faktoren in ihm enthalten und korrekt gewichtet sind.

## Fazit

Die Herausforderung, eine primäre SES früh – also weit vor dem 3. Geburtstag eines Kindes – zu erkennen, wird uns berufs- und wissenschaftsübergreifend weiter begleiten. In Kooperation von Medizin, Public Health, Naturwissenschaften wie Biologie, Neurobiologie, Neuroepidemiologie, Genetik, Psychologie sowie von kognitiven Neurowissenschaften lässt sich diese Herausforderung am besten bewältigen. Vorerst gilt es jedoch, durch wissenschaftliche Forschung Parameter zu erproben, die zur zuverlässigen Früherkennung (und damit auch zur Sekundärprävention), etwa im Rahmen der Kindervorsorgeuntersuchung U5 oder U6, geeignet sein können und eine Akzeptanz über alle genannten Fachgebiete erreichen.

## Footnotes

1 Bei der Delphi-Methode handelt es sich um ein stufenweises Befragungs- und Einschätzverfahren (mehrere Befragungswellen) zu einer bestimmten Thematik, bei dem das Wissen mehrerer anonymisierter Expert*innen aus verschiedenen fachlichen Blickwinkeln zusammengeführt wird. Es können unterschiedliche Ziele verfolgt werden, wie z. B. ein Delphi zur Quantifizierung von Expert*innenmeinungen, ein Vorhersage-Delphi, ein Konsens-Delphi.

2 Die mittlere Diffusivität (MD), eine Variante der MRT sowie das Ausmaß der Anisotropie des Gewebes finden Anwendung in der Diffusionstensorbildgebung (DTI). Der Tensor gibt an, wie stark eine bestimmte Diffusionsrichtung vorliegt; das ist das Maß der „fraktionellen Anisotropie“ (FA).
